# The lure of the lipids: how defensins exploit membrane phospholipids to induce cytolysis in target cells

**DOI:** 10.1038/cddis.2017.69

**Published:** 2017-03-30

**Authors:** Amy A Baxter, Ivan KH Poon, Mark D Hulett

**Affiliations:** 1 Department of Biochemistry and Genetics, La Trobe Institute for Molecular Science, La Trobe University, Melbourne 3086, Victoria, Australia

Direct protein-mediated attack of target cell membranes is a powerful defense mechanism utilized widely throughout nature. Notable examples include pore-forming toxins as well as proteins harboring membrane attack complex/perforin domains.^1, 2^ Another ubiquitously found class of important innate immunity peptides that can mediate membrane permeabilization are cationic antimicrobial peptides (CAPs) of the defensin family, whose mechanism of action is not fully understood. However, recent insights into defensin-mediated membrane disruption indicate that, by recognition of specific phospholipids, certain defensins are triggered to oligomerize, leading to target cell lysis. This has recently been described for several structurally related antifungal defensins of the Solanaceae plant family, including the ornamental tobacco defensin, NaD1, the Australian native tobacco defensin, NsD7 and the tomato defensin, TPP3.^3, 4, 5^ High-resolution crystal structures have revealed that these defensins appear to use their small, conserved disulphide-rich ‘*αβ*' fold in a remarkably efficient and flexible way to specifically recognize phospholipid molecules during innate defense. In the case of both NaD1 and TPP3, these defensins dimerize to form a ‘cationic grip' comprising conserved ‘*β*2–*β*3' loop regions that mediate interaction with the minor yet functionally promiscuous plasma membrane phospholipid, phosphatidylinositol (4,5)-bisphosphate, or PIP2. The binding of PIP2 mediates multimerization of the defensin dimers that has been suggested to induce plasma membrane disruption.^3, 4^ Similarly, NsD7 forms dimers that can assemble into oligomeric complexes with phosphatidic acid (PA) (via interaction with the same *β*2–*β*3 region) as well as lyse PA-containing liposomes, suggesting that this defensin may act via a similar mechanism, albeit by targeting a different lipid.^5^ Indeed, the recognition of lipids by defensins in innate defense is likely conserved across species as we have recently shown that human beta defensin 3 (HBD-3) also binds membrane phospholipids, including PIP2, and can induce cell lysis. Collectively, these lipid-mediated defensin interactions contribute to an emerging niche in the innate immunity peptide field, identifying phospholipids as an important class of membrane targets (Figure 1).

In addition to a role in innate defense, the basis for defensin activity at the plasma membrane has been an area of substantial interest, particularly the potential therapeutic application of defensins with these properties. A previous report by Poon, Baxter, Lay *et al.* demonstrated PIP2-mediated anticancer activity by NaD1 at 10 *μ*M on a range of mammalian tumor cell lines.^3^ Furthermore, PIP2-mediated antitumor activity of TPP3 was also reported, as well as for the evolutionarily unrelated but structurally similar HBD-3, suggesting that PIP2-targeting by these antimicrobial peptides could possibly be exploited as a novel anticancer strategy (Figure 1).^4, 6, 7^ While many cationic antimicrobial peptides, including NaD1 act via direct membrane disruption at low micromolar concentrations, several studies have also demonstrated the ability of CAPs to induce apoptosis in mammalian tumor cells, including via mitochondrial targeting and/or activation of caspases.^8, 9^ Furthermore, whether an innate defense peptide acts via direct membrane targeting (for example, leading to necrosis) or by modifying intracellular pathways (for example, leading to apoptosis), may be dependent on specific experimental parameters, including concentration, treatment duration or target cell type.^8, 9, 10^ Whether, under certain conditions, NaD1 is also capable of activating a programmed cell death pathway such as apoptosis has not previously been investigated and was important to address in light of potential therapeutic applications.

In an article recently published in *Cell Death Discovery*,^11^ we asked the question of whether ‘subacute' treatment of NaD1, that is, under experimental conditions that do not efficiently induce membrane lysis over a short (30 min) treatment time, can induce cell death in mammalian tumor cells via an alternative, non-necrotic mechanism. We reported that over 24 h and across a range of micromolar concentrations below 10 *μ*M, both Jurkat T cells and MM170 melanoma cells displayed considerable growth inhibition but failed to display caspase activation following subacute treatment with NaD1, as demonstrated by a caspase activity assay. In addition, under these subacute treatment conditions cells displayed necrotic phenotypes, including large, stationary plasma membrane blebs and a lack of DNA fragmentation, indicating a necrotic rather than apoptotic response to NaD1. Furthermore, as previously observed under acute experimental conditions for NaD1, TPP3 and HBD-3 (Figure 1), sequestration of plasma membrane PIP2 by neomycin inhibited NaD1-mediated cytotoxicity, under subacute treatment conditions. These data indicate that over a broad range of concentrations and treatment times, NaD1 is a necrosis-inducing agent that functions via a PIP2-targeting mechanism of plasma membrane disruption to elicit cell death. Importantly, since the ability of NaD1 to target mammalian cells had only been investigated previously within narrow experimental parameters (acute concentration of 10μM and only over short time frames up to 30 min), our findings contribute significantly to our understanding of how this defensin mediates cytotoxic activity, thereby furthering the characterization of NaD1 as a membrane-active, phospholipid-targeting peptide.

An important question is whether this phospholipid-targeting mechanism of membrane disruption has been observed beyond a role in host–pathogen interactions. Intriguingly, recent reports on the membrane targeting mechanisms of cell death induction in the pathways of both necroptosis and pyroptosis reveal remarkable parallels with the mechanism described for NaD1 and related defensins.^12, 13, 14^ During both necroptosis and pyroptosis, cells undergo disruption of the plasma membrane, leading to non-apoptotic cell death. This has been shown to occur via targeting of membrane phosphatidylinositol phospholipids, including PIP2 by downstream effector proteins (mixed lineage kinase-like domain, or MLKL in necroptosis, and Gasdermin D, in pyroptosis), with evidence that these proteins may undergo oligomerization at the membrane to facilitate the process of membrane disruption.^12, 13^ It is of considerable interest that these two recently described pathways bear such a striking resemblance to the PIP-targeting mechanism of membrane disruption by NaD1, albeit varying in effector proteins to drive the reactions. Execution of these three pathways, distinct as they are, results in the common outcome of cell death via phospholipid-mediated membrane lysis, suggesting that these may represent examples of convergent evolution, in which PIP2 is a key downstream mediator of cell death.

Collectively, our findings indicate NaD1 and functionally related defensins are phospholipid-targeting peptides that have adapted to target multiple lipids to induce membrane permeabilization and support the existence of a ‘phospholipid code'^15^ that identifies target membranes for defensin-mediated attack as part of a first line of defense across multiple species. Our findings have important implications for our understanding of innate immunity and the structural biology of host–pathogen interactions, as well as for the potential development of novel anticancer therapeutics.

## Figures and Tables

**Figure 1:**
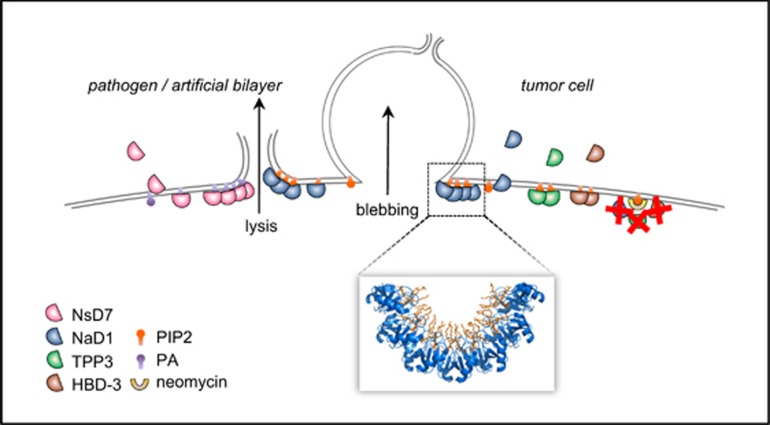
Phopsholipid-binding defensins oligomerize to induce cellular and artificial membrane lysis. Solanaceous plant defensins NaD1 from tobacco (blue) and TPP3 from tomato (green) as well as the human *β*-defensin HBD-3 (brown) have been shown to target PIP2 (orange) in the plasma membranes of pathogens (for example, fungus) and artificial membranes to induce lysis, as well as tumor cells, resulting in blebbing and lysis. The NaD1:PIP2 structure has been solved by X-ray crystallography (PDB code: 4CQK) revealing a large oligomeric complex (inset) that may facilitate membrane destabilization. Similarly, the related tobacco defensin, NsD7 (pink), can oligomerize with PA (purple) and disrupt PA-containing liposomes, suggesting a similar mechanism of membrane permeabilization. By sequestering PIP2 with neomycin (yellow), the activity of NaD1, TPP3 and HBD-3 is inhibited

## References

[bib1] García-Sáez AJ et al J Biol Chem 2011; 286: 37768–37777. 21885440 10.1074/jbc.M111.281592 PMC3199519

[bib2] Dunstone MA, Tweten RK Curr Opin Struct Biol 2012; 22: 342–349. 22658510 10.1016/j.sbi.2012.04.008 PMC3383384

[bib3] Poon IK et al eLife 2014; 3: e01808. 24692446 10.7554/eLife.01808 PMC3968744

[bib4] Baxter AA et al Mol Cell Biol 2015; 35: 1964–1978. 25802281 10.1128/MCB.00282-15 PMC4420927

[bib5] Kvansakul M et al Proc Natl Acad Sci USA 2016; 113: 11202–11207. 27647905 10.1073/pnas.1607855113 PMC5056070

[bib6] Phan TK et al Oncotarget 2016; 7: 2054. 26657293 10.18632/oncotarget.6520 PMC4811302

[bib7] Shafee TM et al Cell Mol Life Sci 2016; 74: 663–682. 27557668 10.1007/s00018-016-2344-5 PMC11107677

[bib8] Mader JS et al Exp Cell Res 2007; 313: 2634–2650. 17570361 10.1016/j.yexcr.2007.05.015

[bib9] Chen J-Y et al Peptides 2009; 30: 2365–2373.19720101

[bib10] Henriques ST, Melo MN, Castanho MA Biochem J 2006; 399: 1–7. 16956326 10.1042/BJ20061100 PMC1570158

[bib11] Baxter AA, Poon IKH, Hulett MD Cell Death Discov 2017; 10: doi: 1038/cddiscovery.2016.102. 10.1038/cddiscovery.2016.102 PMC5253418 28179997

[bib12] Liu X et al Nature 2016; 535: 153–158. 27383986 10.1038/nature18629 PMC5539988

[bib13] Dondelinger Y et al Cell Rep 2014; 7: 971–981. 24813885 10.1016/j.celrep.2014.04.026

[bib14] Quarato G et al Mol Cell 2016; 61: 589–601. 26853145 10.1016/j.molcel.2016.01.011 PMC4769881

[bib15] Baxter A, Hulett M, Poon IK Cell Death Differ 2015; 22: 1893–1905. 26450453 10.1038/cdd.2015.122 PMC4816099

